# Lycopene Reduces the In Vitro Aging Phenotypes of Mouse Oocytes by Improving Their Oxidative Status

**DOI:** 10.3390/vetsci9070336

**Published:** 2022-07-01

**Authors:** Shimaa I. Rakha, Mohammed A. Elmetwally, Hossam El-Sheikh Ali, Ahmed Zaky Balboula, Abdelmonem Montaser Mahmoud, Samy M. Zaabel

**Affiliations:** 1 Department of Theriogenology, Faculty of Veterinary Medicine, Mansoura University, Mansoura 35516, Egypt; shimaaa_ibrahim@yahoo.com (S.I.R.); mmetwally@mans.edu.eg (M.A.E.); helsheikh@mans.edu.eg (H.E.-S.A.); balboula@mans.edu.eg (A.Z.B.); almontaser@mans.edu.eg (A.M.M.); 2 Reproductive Biotechnology Research Laboratory, College of Veterinary Medicine, Mansoura University, Mansoura 35516, Egypt; 3 Animal Sciences Research Center, University of Missouri, Columbia, MO 65211, USA

**Keywords:** lycopene, mouse oocytes, oocyte fragmentation, oxidative stress, postovulatory aging

## Abstract

**Simple Summary:**

Ovulation is the process of oocyte release from the ruptured mature ovarian follicle into the oviduct. Fertilization usually occurs within 10 h post-ovulation in most mammals. If fertilization is delayed, the oocyte viability and quality will decrease, with many deteriorative changes in oocyte phenotype due to oxidative stress. This process is termed postovulatory aging. Postovulatory aging is a major problem that limits the success of many assisted reproductive technologies. Lycopene is a red carotenoid dye found within tomatoes and other fruits and vegetables. Lycopene has been reported to have a strong free-radical scavenging ability. our data showed beneficial effects of lycopene supplementation of in vitro maturation media during in vitro aging of mouse oocytes by reducing the oxidative stress damages that led to their apoptosis. The present study introduces lycopene as a natural supplement to reduce the postovulatory aging of mammalian oocytes.

**Abstract:**

Postovulatory aging is a major problem that limits the success of many assisted reproductive technologies (ARTs). Oxidative stress is a leading cause of oocyte aging. This study investigated the effects of lycopene supplementation of in vitro maturation (IVM) medium during the aging of mouse oocytes on the oocytes’ morphology and oxidative stress status. Mouse cumulus-oocyte complexes (COCs) were collected and cultured in the IVM medium either for 17 h, (freshly matured oocytes), or for 48 h, (in vitro-aged oocytes), with or without lycopene. The rate of fragmented and degenerated oocytes and the oocyte levels of hydrogen peroxide (H_2_O_2_), malondialdehyde (MDA), total antioxidant capacity (TAC), reduced glutathione (GSH), catalase (CAT), and superoxide dismutase (SOD) were estimated and compared. Oocytes aged with 200 nM lycopene revealed significantly less fragmentation and degeneration, lower H_2_O_2_ and MDA levels, and higher TAC, GSH and SOD levels than those aged without lycopene. CAT levels were unchanged by lycopene treatment. Taken together, our data showed beneficial effects of lycopene during in vitro aging of mouse oocytes by reducing the oxidative stress damages that lead to their apoptosis. The present study introduces lycopene as a natural supplement to reduce the postovulatory aging-dependent abnormalities of mammalian oocytes.

## 1. Introduction

Ovulation is the process of oocyte release from the ruptured mature ovarian follicle into the oviduct. Oocytes are usually ovulated encircled by several layers of cumulus cells, collectively known as cumulus-oocyte complexes (COCs) [1]. Ovulated mouse oocytes are arrested at the metaphase of the 2nd meiotic division (MII) until fertilization [2]. Fertilization usually occurs within 10 h post-ovulation in most mammals [3]; however, it may be delayed to 15 h in mice [4,5]. If fertilization is delayed post-ovulation, the oocyte quality will decrease drastically, with many deteriorative changes in oocyte phenotype, a process termed postovulatory aging [6].

Postovulatory aging is known to result in a massive decrease in oocyte quality, with a subsequent decline in the success rates of fertilization in many assisted reproductive technologies (ARTs) [5]. During postovulatory aging, both the quality and viability of oocytes decrease due to many cellular and molecular changes [7]. Postovulatory aging influences the integrity of various oocyte components [8,9]. Moreover, it has been found to be associated with biochemical changes leading to the accumulation of reactive oxygen species (ROS) in mouse oocytes [10,11] Accumulated ROS may lead to cell membrane damage and DNA fragmentation in aged mouse oocytes [12]. In addition, high ROS levels reduce the glutathione (GSH)/glutathione disulfide (GSSG) ratio and accelerates oocyte fragmentation in murine and porcine oocytes [13,14].

Lipid peroxidation has been reported to increase in aging mouse oocytes [8]. Malondialdehyde (MDA) is an end product of polyunsaturated fatty acid peroxidation, and its overproduction is commonly induced by high levels of ROS [15]. Furthermore, elevated MDA levels were reported to be associated with DNA damage and poor outcomes of in vitro fertilization [16,17].

The antioxidant defense system ensures the balance between ROS production and elimination [18]. This system is impaired during oocyte aging due to progressive accumulation of ROS within oocytes [19,20]. Oxidative damage of oocytes has been found to be more severe if the intracellular stores of cellular antioxidants become depleted in a sequence initiated by ROS accumulation [21,22]. Accordingly, the supplementation of antioxidant substances to in vivo and in vitro maturation of mammalian oocytes could reduce this oxidative damage during the postovulatory period. This improvement is a result of a decrease in ROS levels [9] and/or an increase in intracellular antioxidant enzymes, such as superoxide dismutases (SODs) [23] and catalase (CAT) [24].

The total antioxidant capacity (TAC) of the oocyte is the sum of its endogenous and food-derived antioxidants [25]. The reduced glutathione (GSH) is a potent antioxidant substance that presents in ample amounts within all mammalian cells [26]. It has an important role in ROS neutralization [26].

Many antioxidants have been used to improve post-ovulation quality of oocytes. The effect of these antioxidants seems to be dependent on the nature of the substance utilized. L-ascorbic acid and vitamin E were found incapable of preventing oocyte fragmentation during postovulatory aging [27]. Melatonin treatment of in vitro aging mouse oocytes was associated with a lower rate of fragmented oocytes, accompanied by a decline in ROS concentrations compared to counterparts aged without melatonin [20].

Lycopene is a red carotenoid dye found in large concentrations within tomatoes and other fruits and vegetables [28]. Lycopene has been reported to have a strong free-radical scavenging ability [29,30]. Some studies have shown that the addition of lycopene to the in vitro maturation (IVM) medium was associated with improvement of oocyte quality and embryo developmental competence in porcine [31] and bovine oocytes [32,33]. Thus far, the effect of lycopene on the viability of in vitro-aged mouse oocytes has not been examined. Due to the natural origin of lycopene and the improvements in oocyte development observed in other models of in vitro culture, we hypothesized that lycopene could help reduce aging-related abnormalities in postovulatory oocyte aging.

## 2. Materials and Methods

### 2.1. Animals

The current study was conducted at the Reproductive Biology Research Laboratory (RBRL), Department of Theriogenology, Faculty of Veterinary Medicine, Mansoura University, Egypt. The mice used in this study, an albino laboratory-bred strain (BALB/c), were housed in the Medical Experimental Research Center (MERK), Faculty of Medicine, Mansoura University. Mice were maintained in a temperature-controlled environment (21–23 °C) under a standard photoperiod light cycle “12 h light/dark” and provided with feed and water ad libitum. Fifteen mature female mice (8–12 weeks old; ~25 g body weight) were used in this study. Mice were primed two days before ovary retrieval with intraperitoneal injection of 10 IU pregnant mare serum gonadotropin (PMSG, Gonaser^®^, HIPRA, Amer, Spain) [34]. The protocol of this study was permitted by the Committee for Research Ethics at the Faculty of Veterinary Medicine, Mansoura University, Egypt.

### 2.2. Ovary Retrieval and Oocyte Collection

Mice were euthanized by cervical dislocation; ovaries were collected after dissection of the abdominal cavity and washed in pre-warmed (37 °C) sterile physiological saline (0.9% NaCl) [35]. For COC selection and recovery, ovaries were placed in a sterile 60 mm Petri dish containing the handling medium, G-MOPS™ plus (Vitrolife, Göteborg, Sweden).

COCs were collected from the ovaries via gentle puncturing of the antral follicles using a sterile 30-gauge syringe needle under a stereomicroscope (SZ61 zoom, Olympus, Japan) according to Monti and Redi [36]. Collected COCs were examined and classified according to their quality into 3 categories as follows: Category 1 (good quality) oocytes were surrounded by compact multi-layers of follicle cells (corona radiata and cumulus cells) with homogenous, evenly granular ooplasm. Category 2 (fair quality) oocytes were surrounded by only 1–2 layers of cumulus cells with homogeneous ooplasm. Category 3 (poor quality) oocytes had thin or incomplete layers of cumulus cells or were completely devoid of cumulus cells around them (denuded oocytes) and had nonhomogeneous ooplasm. Only good quality COCs were selected and further used for IVM. Denuded and poor-quality oocytes were discarded.

### 2.3. Lycopene Preparation

A stock solution of 1 mM Lycopene (Nawah Scientific Inc., Cairo, Egypt, HIKA2010) was prepared in dimethyl sulfoxide (DMSO; Sigma-Aldrich, D8418), and stored at −20 °C until use. A final concentration of 200 nM was prepared in the maturation medium and used in this study, which was based on previous reports [32,33,37].

### 2.4. In Vitro Maturation (IVM) of Mouse Oocytes

To minimize differences caused by individual variability among animals, oocytes harvested from different mice were pooled and then randomly distributed to different experimental groups. As described by Harada and Kinutani [38], IVM of immature mouse oocytes was performed in a pre-equilibrated IVM medium; Global total (LifeGlobal, Guilford, CT, USA). Briefly, COCs were washed three times in the handling medium, followed by another three washes in the IVM medium. Next, COCs were randomly incubated in groups of 20–25 COCs in 50 µL IVM medium droplets under coverage of mineral oil at 37 °C in 5% CO_2_ in air with maximum humidity (95%) [39]. Based on the experimental design, incubation time was 17 h for the freshly matured group and 48 h for the in vitro-aged groups [20,40].

### 2.5. Evaluation of Oocyte Morphology

After maturation, oocytes from fresh and aged groups were denuded from surrounding cumulus cells by repeated pipetting. Morphological evaluation of oocytes was performed based on calculating the rates of abnormal phenotypes such as fragmentation and degeneration [20,40,41].

### 2.6. Biochemical Estimation of Oxidative and Antioxidative Markers

A sample of 20 oocytes in 100 μL of IVM medium was pelleted by centrifugation at 3000 rpm for 10 min at 4 °C. The pellets were gently rinsed twice with 500 μL of cold phosphate-buffered saline (PBS), and then lysed in 800 μL of lysis buffer (50 mM sodium phosphate, 300 mM NaCl, pH = 8.0) by passing them through repeated freezing and thawing cycles followed by vortexing. Lysed oocytes were centrifuged at 12,000 rpm for 15 min at 4 °C and the supernatant was collected and utilized for biochemical analysis.

The levels of H_2_O_2_ [42] and MDA [43] in the matured oocytes from different groups were calculated colorimetrically using commercially available kits (Biodiagnostics, Egypt) according to the manufacturer’s instructions.

The concentrations of TAC [44], GSH [45], SOD [46], and CAT [47] in the matured oocytes from different groups were calculated using colorimetric assay kits (Biodiagnostics, Cairo, Egypt) per manufacturer’s instructions.

### 2.7. Statistical Analysis

Each experiment was performed at least three times. The differences among the experimental groups were examined using one-way ANOVA followed by Tukey’s multiple comparison test in GraphPad Prism 7 (GraphPad Software, San Diego, CA, USA). Data were represented as the mean ± standard error of the mean (SEM). A receiver operating characteristic (ROC) curve was built using SPSS v.25. Differences of *p* < 0.05 were considered significant.

### 2.8. Experimental Design

Effect of lycopene supplementation to IVM medium on the morphology of in vitro-aged mouse oocytes: A total of 184 prophase I arrested oocytes were divided into three groups based on the time of maturation and presence of lycopene in the maturation medium: fresh (62 oocytes kept in the IVM for 17 h with no lycopene), in vitro-aged (62 oocytes kept in the IVM for 48 h with no lycopene), and in vitro-aged + lycopene (60 oocytes kept in the IVM for 48 h with 200 nM lycopene). Oocyte morphology was assessed in all groups by stereo microscopical examination and fragmentation, and degeneration of oocytes was counted. Abnormal cell rate was calculated in all groups.

To gain further insights about the effect of lycopene supplementation of IVM medium on the levels of oxidative biomarkers in the in vitro-aged mouse oocytes: the previous procedures were sustained, and oocytes from the three groups were collected, lysed, and the concentrations of H_2_O_2_, MDA TAC, GSH, CAT, and SOD were measured and compared among different groups.

## 3. Results

### 3.1. Lycopene Supplementation to IVM Medium Reduces the Aging-Related Morphology Phenotypes in Mouse Oocytes

**Figure 1:**
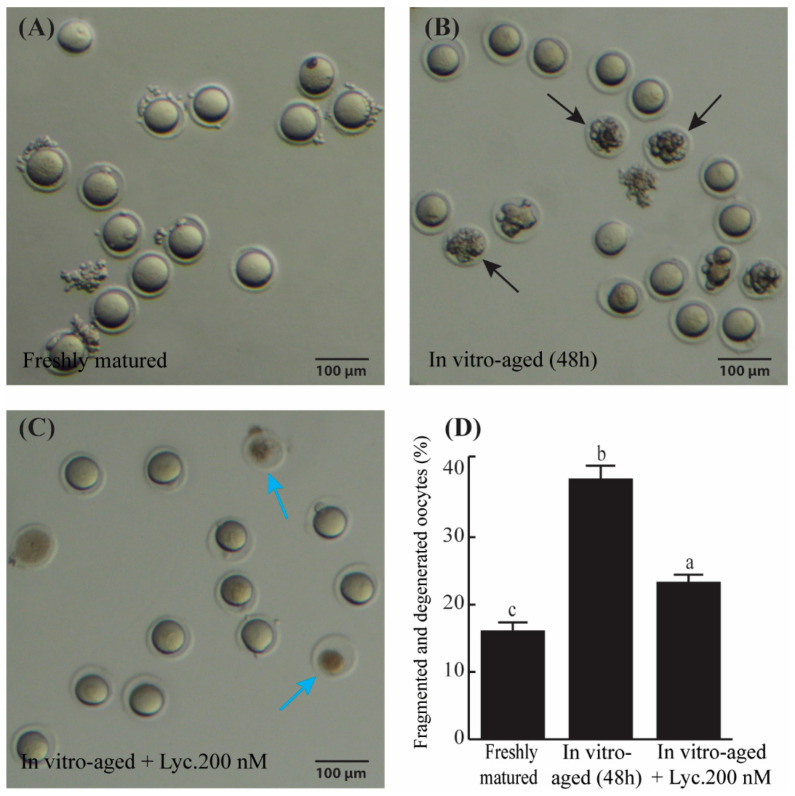
The rate of fragmented and degenerated oocytes in freshly matured, in vitro-aged, and in vitro-aged lycopene-treated groups. (**A**–**C**) Representative photomicrographs of freshly matured oocytes (**A**), in vitro-aged oocytes (**B**) (black arrows denote the fragmented oocytes), and in vitro-aged oocytes treated with 200 nM lycopene (**C)**, (blue arrows refer to degenerated oocytes). (**D**) Quantitative comparison of the rate of oocyte fragmentation between freshly matured, in vitro-aged, and in vitro-aged lycopene-treated groups. Different superscripts indicate significant differences (*p* ˂ 0.05). Scale bars = 100 μm.

Examination of the morphology of in vitro-aged mouse oocytes after 48 h revealed that the percentage of fragmented and degenerated oocytes appeared about 1.7 times greater in aged mouse oocytes incubated without lycopene compared to those aged in the presence of 200 nM lycopene (38.64 ± 1.84% vs. 23.32 ± 0.85%, *p* = 0.0005) (Figure 1). On the other hand, the fragmentation rate of the oocytes significantly increased (*p* ˂ 0.0001) from 16.09 ± 1.13% in the freshly matured group and 38.64 ± 1.84% in the aged group without lycopene (Figure 1D). These results suggest that lycopene helps in reducing aging-related phenotypes in mouse oocytes.

### 3.2. Lycopene Supplementation of IVM Medium Improves the Oxidative Defense Mechanisms in In Vitro-Aged Mouse Oocytes

**Figure 2:**
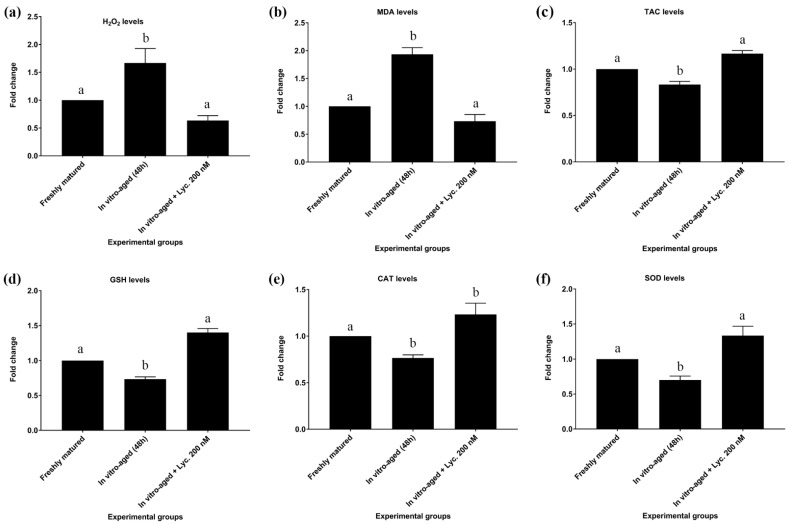
Effect of lycopene addition to IVM medium on oxidative stress and antioxidant biomarker levels of in vitro-aged mouse oocytes. The differences between the experimental groups are shown as fold change relative to the freshly matured oocytes (17 h). The evaluated parameters were as follows: (**a**) Hydrogen peroxide (H_2_O_2_), (**b**) Malondialdehyde (MDA), (**c**) Total antioxidant capacity (TAC), (**d**) Reduced glutathione (GSH), (**e**) Catalase (CAT), and (**f**) Superoxide dismutase (SOD). The error bars represent the standard errors of the means. Different superscripts indicate significant differences (*p* ˂ 0.05).

In vitro-aged mouse oocytes revealed a rise (*p* = 0.018) of about 1.6 times in the levels of H_2_O_2_ compared to freshly matured oocytes (0.270 ± 0.031 mmol/L vs. 0.167 ± 0.009 mmol/L). On the other hand, oocytes aged in the presence of 200 nM lycopene showed a significant decrease (0.170 ± 0.006 mmol/L vs. 0.270 ± 0.031 mmol/L, *p* = 0.02) in levels of H_2_O_2_ in comparison to oocytes aged without addition of lycopene. Their levels of H_2_O_2_ were comparable to those of freshly matured oocytes (Figure 2a).

The levels of MDA in all of the abovementioned groups revealed similar trends to those seen with H_2_O_2_ levels. In vitro-aged mouse oocytes displayed a significant increase of about 1.9 times (8.200 ± 0.503 nmol/mL vs. 4.276 ± 0.203 nmol/mL, *p* = 0.002) in levels of MDA compared to freshly matured oocytes. In contrast, oocytes aged in the presence of 200 nM lycopene revealed a significant decrease in MDA level compared to oocytes aged without lycopene (5.700 ± 0.586 nmol/mL vs. 8.200 ± 0.503 nmol/mL *p* = 0.02), but their MDA levels did not differ significantly (5.700 ± 0.586 nmol/mL vs. 4.276 ± 0.203 nmol/mL, *p* = 0.15) from those of freshly matured oocytes (Figure 2b; Table S1).

The TAC levels of in vitro-aged mouse oocytes revealed a modest, though significant (0.513 ± 0.009 mmol/L vs. 0.593 ± 0.020 mmol/L, *p* = 0.025) decrease compared to TAC levels of freshly matured oocytes. On the other hand, oocytes aged in the presence of 200 nM lycopene revealed a significant increase in TAC levels in comparison to oocytes aged without lycopene (0.590 ± 0.015 mmol/L vs. 0.513 ± 0.009 mmol/L, *p* = 0.029). However, their TAC levels were equivalent to those of freshly matured oocytes (Figure 2c; Table S1).

In addition to TAC, levels of GSH, CAT, and SOD showed the same pattern after inducing the in vitro aging. All of them significantly decreased by about 30% after inducing the aging in absence of lycopene supplementations. On the other hand, lycopene supplementation enhanced the antioxidant activity of the aged oocytes to be comparable to the non-aged fresh group as summarized in Figure 2 and Table S1.

Regarding GSH levels, in vitro-aged mouse oocytes without lycopene showed a significant decrease in levels of GSH compared to freshly matured oocytes (2.713 ± 0.056 mmol/L vs. 3.717 ± 0.123 mmol/L, *p* = 0.0004). Conversely, oocytes aged in the presence of 200 nM lycopene showed a significant increase in GSH levels relative to in vitro-aged mouse oocytes without lycopene (3.693 ± 0.063 mmol/L vs. 2.713 ± 0.056 mmol/L, *p* = 0.0005). However, their GSH levels were insignificantly different (3.693 ± 0.063 mmol/L vs. 3.717 ± 0.123 mmol/L, *p* = 0.98) from those of freshly matured oocytes (Figure 2d; Table S1).

Concerning CAT levels, in vitro-aged mouse oocytes without lycopene exhibited a significant decline in levels of CAT compared to freshly matured oocytes (0.217 ± 0.009 U/L vs. 0.293 ± 0.003 U/L, *p* = 0.008). On the other hand, CAT levels revealed a tendency to increase in oocytes aged in the presence of 200 nM lycopene compared to in vitro-aged mouse oocytes without lycopene (0.263 ± 0.018 U/L vs. 0.217 ± 0.009 U/L, *p* = 0.06). No significant difference (0.263 ± 0.018 U/L vs. 0.293 ± 0.003 U/L, *p* = 0.2) in CAT levels was detected between oocytes aged in the presence of 200 nM lycopene and freshly matured oocytes (Figure 2e; Table S1).

In regard to SOD levels, in vitro-aged mouse oocytes without lycopene revealed a significant decrease in SOD levels compared to freshly matured oocytes (245.333 ± 17.285 U/mL vs. 346.000 ± 1.000 U/mL, *p* = 0.002). On the other hand, oocytes aged in the presence of 200 nM lycopene revealed a significant increase in SOD levels compared to in vitro-aged mouse oocytes without lycopene (327.000 ± 9.292 U/mL vs. 245.33 3± 17.285 U/mL, *p* = 0.005), but did not differ significantly (327.000 ± 9.292 U/mL vs. 346.000 ± 1.000 U/mL, *p* = 0.5) from SOD levels of freshly matured oocytes (Figure 2f; Table S1).

### 3.3. ROC Curve Analysis of In Vitro-Aged Mouse Oocytes

**Figure 3:**
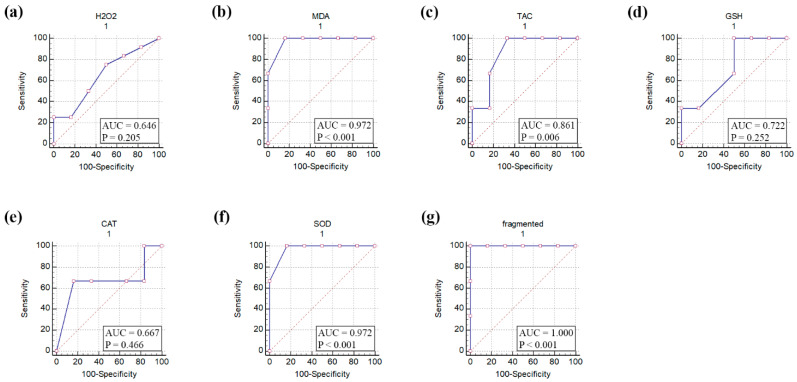
Receiver operating characteristic (ROC) curve of oxidative stress biomarkers, antioxidant biomarkers, and fragmentation of in vitro-aged mouse oocytes. (**a**) Hydrogen peroxide (H_2_O_2_), (**b**) Malondialdehyde (MDA), (**c**) Total antioxidant capacity (TAC), (**d**) Reduced glutathione (GSH), (**e**) Catalase (CAT), (**f**) Superoxide dismutase (SOD), and (**g**) fragmented oocytes. AUC, area under the curve. *p* values ˂ 0.05 indicate significance.

The ROC curve analysis of seven parameters that included H_2_O_2_, MDA, TAC, GSH, CAT, SOD, and oocyte fragmentation in aged mouse oocytes suggested MDA, TAC, SOD, and oocyte fragmentation to be significantly affected by in vitro aging of mouse oocytes (Figure 3).

## 4. Discussion

Post-ovulatory aging of mammalian oocytes is a process that severely affects developmental competence by reducing the rates of oocyte survival, sperm penetration during fertilization, and embryo development [48]. In this study, we were able to reduce the severity of abnormal phenotypes associated with oocyte aging by supplementation of the IVM medium with lycopene. Fragmentation and degeneration of the oocytes were significantly reduced after lycopene supplementation to a level comparable to that of the fresh group. Other studies revealed that antioxidant supplementation improves oocyte quality and reduces fragmentation rates. An increase in the number of degenerated and fragmented mouse oocytes after 12 h and 24 h of their in vitro aging was seen in oocytes untreated compared to oocytes treated with quercetin, which is a plant pigment (flavonoid) known for its antioxidant activity [40]. Another compound, resveratrol, a natural phenolic compound of plant origin, was found to decrease the apoptosis rate of mouse oocytes aged in vivo compared with the non-resveratrol treated group [49]. Furthermore, melatonin supplementation significantly reduced the percentage of acquired abnormal morphology of mouse oocytes over time compared to control aged mouse oocytes [20]. In porcine aged oocytes, melatonin supplementation showed normal morphology with significantly low fragmentation percentage after 48 h aging [41].

Postovulatory aging changes are mainly attributed to increased ROS production levels, apoptosis, and oocyte fragmentation [8,50,51,52]. Therefore, the supplementation of oocytes with exogenous antioxidants post-ovulation or after IVM (before fertilization) can be considered an effective approach to reduce the oocyte damage brought by ROS during aging [6]. Some studies have shown that lycopene has an important effect on antioxidant biomarker levels. Mackinnon and Rao [53] showed that supplementation of lycopene by post-menopausal women for 4 months significantly increased serum TAC and decreased lipid peroxidation. However, there is no significant effect on any of the antioxidant enzymes CAT, SOD, or GPx. Additionally, Velmurugan and Bhuvaneswari [54] revealed that lycopene decreased oxidative injury by stimulating the levels and activities of antioxidant enzymes, including glutathione (GSH), glutathione-S-transferases, and glutathione peroxidase, in animals with gastric cancer. Moreover, it was found that lycopene treatment showed protection against gastric ulcers in rats through its antioxidant effects, as SOD activity and GSH levels were higher in the lycopene-treated group. However, CAT activity and MDA levels were lower in the lycopene-treated group when compared with controls. [55]. Additionally, lycopene can give protection against experimental esophagitis via increasing GSH levels and SOD and CAT enzyme activities, and decreasing MDA levels in the lycopene-treated group when compared with controls [56].

We then tried to understand how lycopene reduced aging abnormalities. A strong explanation for this improving effect could be due to the direct effects on the oocyte redox status. With lycopene supplementation, we found a decrease in the oocyte levels of H_2_O_2_ and MDA and an increase in the oocyte levels of TAC, GSH, CAT, and SOD. This may support another study in porcine oocytes that showed a decrease in H_2_O_2_ levels, and an increase in GSH concentrations, as well as CAT and SOD expression levels after supplementation of 2.5 μM astaxanthin, a ketocarotenoid with structural similarities to lycopene, during their in vitro aging for 42–44 h [57]. In addition, in bovine oocytes, lycopene supplementation to the IVM medium led to a significant reduction in ROS levels [37]. In aged porcine and mouse oocytes, reduced ROS levels have also been reported after melatonin supplementation during their in vitro aging at concentrations of 1 mM and 2 mM, respectively [41,58]. The ROS scavenging effect of melatonin and other antioxidants mentioned above was accompanied by normal mitochondrial distribution within the cytoplasm of oocytes. Our study strongly suggests that lycopene has a similar promising and preventive effect on in vitro-aged oocytes.

Lipid peroxidation has been reported to be increased in aging mouse oocytes [8]. MDA is an end product of polyunsaturated fatty acid peroxidation, and its overproduction is induced by high levels of ROS [15]. In the present study, lycopene supplementation decreased MDA significantly in the aged oocytes. This finding suggests a role for lycopene in preserving the structural integrity of polyunsaturated fatty acids within oocytes. Consistent with our observations, a better distribution of lipid droplets has been observed in bovine oocytes after lycopene supplementation [37].

Postovulatory aging of oocytes initiates different developmental programs, such as fragmentation, programmed cell death, and abnormal development [59]. The reasons for oocyte fragmentation during their aging are still poorly understood [48,60]. However, accumulating evidence suggests that fragmentation of aged murine oocytes is a consequence of alterations in the protein expression levels of oocyte pro- and anti-apoptotic factors [59]. Members of the Bcl-2 gene family including Bcl2 and Bax, have been implicated in this process through activation of effector caspases which in turn execute cell death [59,61]. In line with these observations, ROC curve analysis of aged mouse oocytes conducted by the present study pointed to the number of fragmented oocytes together with the levels of MDA, TAC, and SOD as specific markers for oocyte aging.

This study is the first to report that lycopene can improve the quality of aged oocytes by reducing fragmentation rates. However, further study is warranted to assess the effect of lycopene on in vitro fertilization and embryo production of aged oocytes.

## 5. Conclusions

Our study has demonstrated that lycopene as an antioxidant successfully maintained the morphology and alleviated oxidative stress during in vitro aging of mouse oocytes by preventing oxidative damages that lead to aging and apoptosis. The present study introduces lycopene as a natural supplement to control the postovulatory aging of mammalian oocytes processed for clinically assisted reproductive technology.

## Data Availability

Not applicable.

## Supplementary Materials

The following supporting information can be downloaded at: https://www.mdpi.com/article/10.3390/vetsci9070336/s1, Table S1: Effect of lycopene addition to IVM medium on oxidative stress and antioxidant biomarkers levels of in vitro-aged mouse oocytes.
