# Supplementary material for: Lycopene Reduces the In Vitro Aging Phenotypes of Mouse Oocytes by Improving Their Oxidative Status
Source: Vet Sci. 2022 Jul 1;9(7):336. doi: 10.3390/vetsci9070336 (PMC9324547; doi:10.3390/vetsci9070336)
Supplement: Supplementary file 1 [file vetsci-09-00336-s001.zip › vetsci-1772276-supplementary.pdf]

Table S1. Effect of lycopene addition to IVM medium on oxidative stress and antioxidant biomarkers levels of in vitro aged mouse oocytes.

Item	Fresh (Matured for 17 h)	In vitro aged (Matured for 48 h)	In vitro aged (Matured for 48 h + Lyc. 200 nM)
H_2O_2 (mmol/L)	0.167 ± 0.009 [a]	0.270 ± 0.031 [b]	0.170 ± 0.006 [a]
MDA (nmol/mL)	4.276 ± 0.203 [a]	8.200 ± 0.503 [b]	5.700 ± 0.586 [a]
TAC (mmol/L)	0.593 ± 0.020 [a]	0.513 ± 0.009 [b]	0.590 ± 0.015 [a]
GSH (mmol/L)	3.717 ± 0.123 [a]	2.713 ± 0.056 [b]	3.693 ± 0.063 [a]
CAT (U/L)	0.293 ± 0.003 [a]	0.217 ± 0.009 [b]	0.263 ± 0.018 [b]
SOD (U/mL)	346.000 ± 1.000 [a]	245.333 ± 17.285 [b]	327.000 ± 9.292 [a]

[1] Different superscript letters in the same row indicate significant differences ($P < 0.05$). Data are presented as mean ± SEM.
